# Role of White Blood Cells in Blood- and Bone Marrow-Based Autologous Therapies

**DOI:** 10.1155/2018/6510842

**Published:** 2018-07-10

**Authors:** William King, Krista Toler, Jennifer Woodell-May

**Affiliations:** Zimmer Biomet, 56 East Bell Drive, Warsaw, IN 46580, USA

## Abstract

There has been significant debate over the role of white blood cells (WBCs) in autologous therapies, with several groups suggesting that WBCs are purely inflammatory. Misconceptions in the practice of biologic orthopedics result in the simplified principle that platelets deliver growth factors, WBCs cause inflammation, and the singular value of bone marrow is the stem cells. The aim of this review is to address these common misconceptions which will enable better development of future orthopedic medical devices. WBC behavior is adaptive in nature and, depending on their environment, WBCs can hinder or induce healing. Successful tissue repair occurs when platelets arrive at a wound site, degranulate, and release growth factors and cytokines which, in turn, recruit WBCs to the damaged tissue. Therefore, a key role of even pure platelet-rich plasma is to recruit WBCs to a wound. Bone marrow contains a complex mixture of vascular cells, white blood cells present at much greater concentrations than in blood, and a small number of progenitor cells and stem cells. The negative results observed for WBC-containing autologous therapies* in vitro* have not translated to human clinical studies. With an enhanced understanding of the complex WBC biology, the next generation of biologics will be more specific, likely resulting in improved effectiveness.

## 1. Introduction

There has been recent significant debate on the role of white blood cells (WBCs) in autologous therapies in the orthobiologics literature [1–6]. Unfortunately, after more than 15 years of clinical use of autologous therapies, there are few randomized and controlled clinical trials (RCTs) and even fewer RCTs which also characterize the cellular content of the autologous therapy for any orthopedic disease or injury. RCTs are critical to the advancement of research in this space because of the propensity for large placebo responses when a therapy is perceived to be “novel” [7]. In the absence of level 1 clinical evidence, most of the debate has been centered on the varying options for concentrating cells, platelets, and proteins [8–11], with few studies correlating the concentrations of these components with cell culture or clinical outcomes. Furthermore, there are few* in vitro* studies that have been shown to correlate to clinical results in orthopedics [12]. Key misconceptions arising from these preclinical studies are that platelets only contain growth factors, WBCs are purely inflammatory, and bone marrow contains only stem cells. This review will address these key misconceptions in the field of autologous therapies and address steps that should be taken to advance the development of orthobiologics.

## 2. Platelets Not Only Contain Growth Factors but Also Recruit WBCs to Damaged Tissue

In the field of autologous therapies, platelet-rich plasma (PRP) is a catch-all term that refers to a fraction of blood containing a higher concentration of platelets than whole blood. There are commercially available devices which prepare PRP in different ways. Devices that isolate the buffy coat in a small volume of plasma generally produce PRP containing high concentrations of platelets and WBCs, known as leukocyte-rich PRP (L-PRP). Other devices process blood at lower centrifugal forces and isolate lower concentrations of platelets and very few WBCs. This composition is often referred to as pure PRP (P-PRP) [13]. Platelets are known in orthopedic literature for containing tissue healing growth factors like transforming growth factor-beta (TGF-*β*), platelet derived growth factor (PDGF), and insulin-like growth factor-1 (IGF-1) [14]. One could intuitively think that increasing the concentration of platelets would result in similarly increased concentrations of growth factors, but experimental evidence has not proven this relationship [15]. Mixing, resuspension, and pipetting aliquots of samples may contribute variability that has made it challenging to discern this relationship. Although platelets are known for containing tissue healing growth factors, they contain many other proteins that could play a role in tissue healing.

**Figure 1:**
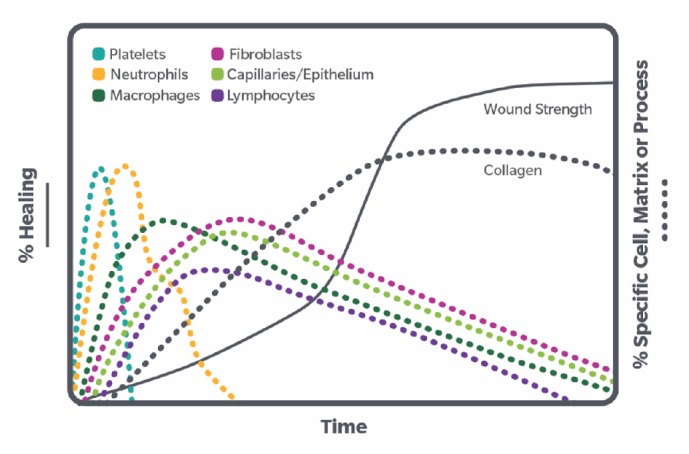
Role of different cell types, including WBC subtypes, in wound repair (adapted from [21]).

Platelets provide hundreds of bioactive proteins whose key roles include hemostasis and inducing recruitment of WBCs [16]. Inflammation is part of the wound healing process [17]. In the first step of tissue repair, platelets arrive at a wound site, degranulate, and release their diverse contents, inclusive of growth factors. Platelets provide proteins for primary and secondary hemostasis including fibrinogen and von Willebrand factor. Once bound to extracellular matrix at a wound site, activated platelets secrete a wide variety of chemokines which attract WBCs, including CXCL4 and CXCL7, which are the most abundant chemokines in platelets. Both of these chemokines play a role in neutrophil recruitment and binding to a wound site, among many other functions. Activated platelets also have p-selectin on their surface which binds circulating WBCs [18]. Together, these well-characterized steps in the wound healing process indicate that platelets contain numerous factors to recruit WBCs to damaged tissue and retain WBCs to induce repair (Figure 1).

When applied to damaged tissue, P-PRP will recruit WBCs, including polynuclear neutrophils, to repair the damage [22, 23]. For example, when PRP containing 588 x 10^3^/mm^3^ platelets and 0.10 x 10^3^/mm^3^ WBCs was applied to wounds in rats, significantly increased inflammation and wound repair were observed compared to saline controls [23]. In a separate study, leukocyte-reduced PRP increased cellular infiltration into canine radial ostectomy gap compared to saline control [24]. Leukocyte-reduced PRP also induced the infiltration of inflammatory cells into collagen sponges used in canine anterior cruciate ligament repair [25]. These data indicate that the one of the core functions of platelets in PRP is to recruit WBCs which aid in tissue repair.

## 3. The Behavior of WBCs Is Adaptive

The essential role of WBCs in wound healing and tissue repair has been established for decades [26], and new discoveries surrounding macrophage and T-cell polarization have generated recent excitement [27]. WBC function is adaptive, depending on the environment. For example, WBCs can be inflammatory in a manner that hinders tissue repair under certain conditions such as an active infection, whereas they can induce tissue repair during normal healing processes [28]. Even so, WBC-rich concentrated bone marrow aspirate (cBMA) has been used to address infected bone nonunions in the absence of antibiotics, further supporting the versatility of WBC behavior in challenging environments [29]. The many roles of WBCs that might be performed in autologous therapies have not been accurately communicated to the orthopedic community.

### 3.1. The Role of Macrophages

The biology of WBCs is complex, as they are involved in both inflammation and tissue healing. Macrophages are monocytes that have marginated into tissue, and these macrophages can have varying phenotypes, including M1 and M2 macrophages [30–32]. Recent research into macrophage maturation has demonstrated that macrophages polarize into classic and alternative subtypes, or M1 or M2. Classic M1 macrophages are inflammatory cells that have microbicidal activity and produce interferon gamma (IFN*γ*). Recent research has shown an alternative activation pathway, resulting in M2 macrophages, which perform immunoregulation, matrix deposition, and tissue remodeling roles [33]. Intrapatellar fat pads from patients with OA showed a higher percentage of M2 macrophages than subcutaneous fat or anterior cruciate ligament tissue and were able to better inhibit catabolic processes in the cartilage [34]. Clinical research has shown that both M1 and M2 states are activated in the synovial lining of OA patients, leading to a chronic inflammatory state [30]. Autologous anti-inflammatory therapies designed to promote M2 polarization and ameliorate this chronic inflammatory state could potentially lead to sustained OA pain relief. A customized PRP has been prepared by removing TGF-*β*1 using neutralizing antibodies, as TGF-ß1 can cause fibrosis and inhibit optimal muscle healing. This PRP recruited more M2 macrophages to a murine muscle injury model than unmodified PRP and untreated control [35]. Future research will be required to determine if clinical benefits observed with autologous point-of-care therapies are related to macrophage polarization.

T-cells are WBCs with varying subtypes which also have been hypothesized to have a role in osteoarthritis. For example, T_H_2 cells can secrete IL-17, which can induce chemokine release from chondrocytes and synoviocytes contributing to OA progression [36]. Alternatively, regulatory T (Treg) cells have been shown to inhibit the function of IFN-*γ* and tumor necrosis factor alpha (TNF-*α*) and protect tissue engineering cartilage in vitro [37]. Many different T-cell phenotypes have been characterized in the blood, synovial fluid, and synovial tissue of OA patients, and there is evidence that some are involved in the pathogenesis of OA, including Th1 cells, Th9 cells, Th17 cells, Treg cells, cytotoxic T-cells, and Tm cells. However, a causal relationship between T-cell phenotype and OA progression has not been established [38]. Future research into the role of T-cells in autologous therapies could enable a unique mechanism for tissue repair. In summary, the concept that WBCs are “bad” does not reflect the diverse cell types and functions of WBCs.

## 4. Bone Marrow Concentrate Contains a Complex Mixture of Cell Types

Bone marrow contains a complex mixture of cell types, including hematopoietic stem cells (HSCs) [39], which form the blood system, mesenchymal stem cells (MSCs) [40], which form connective tissues, endothelial progenitor cells (EPCs) [41], which form the vascular system, WBC subtypes of varying stages of maturation, red blood cells of varying stages of maturation, and platelets. Caplan and colleagues have reported that 1:10,000 cells in bone marrow are MSCs in a newborn, and this proportion declines with age [42]. Weissman and colleagues have reported that approximately 0.08% of bone marrow mononuclear cells are HSCs in young adults and approximately 0.25% of bone marrow mononuclear cells are HSCs in elderly adults [43]. Taylor and colleagues have reported the frequency of EPCs in bone marrow to be 0.007% of the total nucleated cells [44]. If bone marrow concentrates and variations thereof have a therapeutic effect in orthopedics, then the >99% of white blood cells in bone marrow which has nonstem or progenitor cell phenotypes would likely play a significant role.

Bone marrow transplants have been used since 1956 to treat leukemias, lymphoproliferative disorders, and nonmalignant disorders [45]. Autologous red blood cell-reduced cBMA has been explored in a wide variety of orthopedic [46], vascular [47, 48], and cardiovascular conditions [49]. There have also been efforts to isolate and concentrate more specific cell types from autologous bone marrow. For example, CD34^+^ cells have been isolated by magnetic bead sorting and explored for the treatment of critical limb ischemia [50]. Despite the assertion that WBCs are disadvantageous in autologous therapies due to their inflammatory properties, these applications of WBC-rich cBMA have had favorable safety profiles, although the efficacy signals have been mixed across indications.

Characterization of cBMA and its variants may lead to the pursuit of more specific indications for the devices used to produce these cellular therapies. Recent characterization of cBMA produced using a tuned buoy system has demonstrated complex cellularity, including 233 ± 61 k/*μ*l total nucleated cells, a platelet concentration of 753 ± 233 k/*μ*l, and 3,274 ± 2,159 CFU-F/ml [19]. This formulation has also been characterized to contain high concentrations of anti-inflammatory cytokines, including IL-1ra (73,978 ± 39,464 pg/ml) and sTNF-RII (3,932 ± 1,301 pg/ml) and low concentrations of inflammatory cytokines, including IL-1*β* (14.5 ± 11.4 pg/ml) and TNF*α* (1.4 ± 0.7). In baseline bone marrow aspirate and cBMA, there was significant and strong correlation between WBCs and IL-1ra concentration (R^2^ = 0.92, p = 0.0002) [51]. In general, bone marrow and bone marrow concentrates contain greater concentrations of WBC than baseline blood or PRP (Table 1).

### 4.1. Comparison of In Vitro Results and Clinical Outcomes

There have been several* in vitro* and device output experiments that have suggested WBCs are purely inflammatory in the context of autologous therapies [3, 5, 52–55]. In a PRP characterization study, there was significant correlation reported between MMP-9 and neutrophils, IL-1*β* and neutrophils, and IL-1*β* and monocytes [55]. In another* in vitro* study, cultured fibroblast-like synoviocytes expressed more MMPs and inflammatory cytokines when cultured with L-PRP compared to platelet-poor plasma [54]. In a tendon-PRP study, when equine tendon explants were cultured with L-PRP and P-PRP, explants cultured with L-PRP had greater gene expression of IL-1*β* and TNF*α* [3]. To date, these* in vitro* outcomes have not translated to human clinical results. For example,* in vitro* data for culturing tendon cells with L-PRP is negative [54], but positive outcomes of L-PRP injections for chronic lateral epicondylitis have been reported clinically [56–58]. Further, in contrast to the* in vitro* studies demonstrating inflammatory characteristics of leukocytes, a WBC-containing formulation has demonstrated anti-inflammatory properties* in vitro* [59–61]. There are several possibilities which could explain these discrepancies. For example, L-PRP often has more red blood cells than P-PRP which could suffocate monoculture layer [62] unless appropriate cell culture well inserts are used [61]. Also, cell culture experiments have been designed and conducted without matching donors between culture layer and WBC-containing PRP [4], likely leading to a mismatch of antigen-presenting cells and consequent inflammatory reaction. Furthermore, many of the papers suggesting WBCs are inflammatory only measure pg/ml concentrations of IL-1 [55] and do not report the concentration of IL-1ra (a key anti-inflammatory cytokine which inhibits IL-1 signaling) in WBC-containing autologous therapies, which are typically in the ng/ml concentration range [63].

### 4.2. White Blood Cells Produce Anti-Inflammatory Cytokines

Studies have shown that IL-1ra is secreted from monocytes and neutrophils [17] and therefore formulations that reduce WBCs will reduce IL-1ra content. IL-1ra is found predominantly inside the cells and not on the membranes or in the plasma [18] which enables them to serve as delivery vehicles* in vivo*. In early clinical studies, WBC concentration has been shown to strongly correlate with the concentration of IL-1ra in different autologous therapies [51, 64]. Bone marrow, which contains greater concentrations of WBCs than whole blood, contains high levels of IL-1ra [65]. P-PRP does not have high concentrations of IL-1ra because it does not contain WBCs [66]. Therefore, when conducting characterization studies of autologous point-of-care therapies, it is important to characterize both sides of a biological process (i.e., inflammatory/anti-inflammatory or anabolic/catabolic).

Blood and bone marrow are not fundamentally different solutions, but rather a continuation of the same biological system. All blood cells have a similar life history: they are generated from a common stem cell in the bone marrow. They differentiate to form the different blood cell types and osteoclasts. WBCs principal functions are not limited to actions in the blood stream [67]. Therefore, when evaluating the composition of an autologous therapy, concentrating WBCs from bone marrow cannot be good for many orthopedic applications while concentrating WBCs from blood are bad for the same orthopedic applications.

To date, initial studies with both L-PRP and P-PRP have suggested pain reduction in intra-articular injections of OA [68–77]. The only head-to-head trial of L-PRP and P-PRP in osteoarthritis showed no difference in pain relief [70]. A meta-analysis of PRP OA studies showed that there was no difference in incidence of adverse events between L-PRP and P-PRP [1]. A large case series (n = 840) of WBC-rich cBMA injections for OA has demonstrated a favorable safety profile [78] and pain relief [79]. In a prospective, single-blind, placebo trial, 25 patients with bilateral knee pain were randomized to receive cBMA into one knee and saline placebo into the other knee. cBMA had a favorable safety profile, but there were similar degrees of pain relief in both cBMA- and saline-treated knees, indicating that further research may be necessary to determine the role of bone marrow WBCs in potentially relieving OA pain [80]. There have not been randomized and controlled trials comparing cBMA with other intra-articular therapies. Also, serum produced by stimulated WBCs clotted on glass beads had positive clinical outcomes in orthopedics [81–84]. However, the clinical results and cytokine concentrations obtained have not been repeatable in several subsequent studies [85, 86]. Randomized, placebo-controlled, sufficiently powered trials will be required to provide definitive evidence on the safety and effectiveness of these autologous therapies with different WBC concentrations.

## 5. Future Direction

Developers of autologous point-of-care therapies should start with a fundamental understanding of the disease state and what potential cells and proteins could address the disease. Although* in vitro* studies can provide important mechanism of action data, an emphasis on trying to find a large animal model with a naturally occurring version of a human disease could be more useful at determining the efficacy of a potential therapy. Finally, a clear strategy towards developing a clinical evidence plan that allows for demonstrating the safety and efficacy of the therapy should involve conversations with the many stakeholders involved in medical device development. As an example, there has been recent interest in the content of exosomes produced by cultured bone marrow-derived stem cells and their potential therapeutic applications [87]. Future research on exosomes and unique cellular fractions in cBMA may provide insights into its therapeutic potential.

With current devices it is challenging to perform dosing studies, as every patient's tissues contain different concentrations of platelets, cells, and proteins. Furthermore, the concentration of these factors can vary with the time of day within patients, with natural interpatient variability, and with patient comorbidities. Currently, there are no autologous therapies which can output a normalized number of platelets or cells (i.e., 10 million WBCs). However, it is not clear that even if that were possible, if normalizing cell or platelet numbers would lead to greater standardization, as patient's comorbidities can lead to cells having different functional capacities. Current regulatory structure in the United States will require these development programs to potentially take 8-10 years or longer and 30-50 million dollars [12]. Careful market analysis will allow researchers to determine if there is sufficient opportunity to justify the preclinical and clinical investment needed to obtain the indication to treat the target disease. Regulatory approval is not enough for a point-of-care therapy to be a successful product. Although many aspects determine the commercialization of a medical device, reimbursement is a key driver of a product's ultimate commercial success. The development of the clinical evidence plan should include conversations with payers so one does not end up with regulatory approval but no mechanism to get the product paid for (i.e., FDA approval but no insurance coverage). In summary, new devices to make autologous point-of-care therapies will need to address large unmet medical needs in a manner that takes into account the realities of the changing healthcare landscape.

## Figures and Tables

**Table 1:** Concentration of WBC, platelets (PLT), and red blood cells (RBC) in baseline blood, bone marrow, and devices which process these tissues differently (adapted from previously published from laboratory experiments [19, 20]).

**Autologous Product Type:**	**WBC (k/** ***μ*** **l)**	**PLT (k/** ***μ*** **l)**	**RBC (M/** ***μ*** **l)**
Whole Blood	5.4 ± 1.8	175 ± 70	5.5 ± 1.1

PPP	0.0 ± 0.0	45 ± 19	0.0 ± 0.0

Conditioned Serum with Short Glass Bead Incubation	0.0 ± 0.0	11 ± 2	0.0 ± 0.0

Conditioned Serum with Long Glass Bead Incubation	0.0 ± 0.0	14 ± 6	0.0 ± 0.0

Concentrated PPP	0.0 ± 0.0	55.0 ± 14.7	0.0 ± 0.0

Leukocyte-Reduced PRP	2.4 ± 2.1	533 ± 235	0.4 ± 0.2

Leukocyte-Enriched PRP	28.1 ± 6.9	1,745 ± 439	0.9 ± 0.3

Autologous Protein Solution	46.5 ± 14.0	707 ± 444	1.5 ± 1.1

Bone Marrow Aspirate	23 ± 7	117 ± 26	4 ± 1

Concentrated Bone Marrow Aspirate	233 ± 61	753 ± 233	3 ± 2
